# Is there a right place? The effect of within-leaf clutch location on offspring survival in a glassfrog

**DOI:** 10.1371/journal.pone.0309642

**Published:** 2025-04-11

**Authors:** Francesca N. Angiolani-Larrea, Anyelet Valencia-Aguilar, Marina Garrido-Priego, Mélissa Peignier, Jaime Culebras, Lelis Jindiachi, José G. Tinajero-Romero, Juan M. Guayasamin, Eva Ringler

**Affiliations:** 1 Division of Behavioural Ecology, Institute of Ecology and Evolution, University of Bern, Bern, Switzerland; 2 Institute of Animal Physiology, Department of Animal Physiology and Molecular Biomedicine, Justus-Liebig-University Giessen, Giessen, Germany; 3 Photo Wildlife Tours, Quito, Ecuador; 4 Fundación Cóndor Andino, Quito, Ecuador; 5 Pueblo Shuar Arutam, Federación Interprovincial Centros Shuar (FICSH), Sucúa, Ecuador; 6 Escuela de Biologia, Universidad de Costa Rica, San José, Costa Rica; 7 Universidad San Francisco de Quito USFQ, Laboratorio de Biología Evolutiva, Colegio de Ciencias Biológicas y Ambientales COCIBA, Instituto BIÓSFERA-USFQ, Campus Cumbayá, Quito, Ecuador; 8 Tandayapa Cloud Forest Station, Universidad San Francisco de Quito USFQ, Quito, Ecuador; Central University of South Bihar, INDIA

## Abstract

The choice of where to breed can have fundamental consequences for offspring development and survival. Among amphibians, desiccation is one of the biggest threats to offspring survival, especially in species that deposit their clutches in terrestrial habitats. In several species, hydration of the clutch is ensured by a caregiving parent, but in species without prolonged care, site selection becomes critically important for securing constant external sources of hydration. We used the Spiny Cochran frog (*Teratohyla spinosa*), a Neotropical glassfrog in which females perform only short-term brooding to clutches, but then both parents leave the offspring, to test the effect of oviposition site selection within leaves on offspring development and survival. Previous observations have revealed that this species preferentially deposits eggs on the underside of leaves close to their margins. We hypothesized that *T. spinosa* strategically chooses this position to ensure clutch hydration during embryonic development, as water drops will slide from the edges to the tip of the leaves before dripping. To this end, we performed a clutch translocation experiment where we manipulated the location of clutches by placing them away from the leaf margin and compared their level of hydration, hatching time, and mortality rate to clutches that were kept on the leaf margins. Contrary to our expectations, we found that clutch hydration and mortality were not affected by the location on the leaf. These findings suggest that the observed clutch deposition on the edges of leaves in this species is not enhancing hydration conditions – at least under high humidity conditions.

## Introduction

In many animal species, the choice of where to breed can have strong impacts on offspring survival and development [1,2]. These decisions are often non-random and influenced by factors such as intraspecific competition, habitat structure, predation pressure, and climatic variables [2]. Therefore, individuals are expected to trade-off the allocation of resources to their current or future offspring, and their own needs [1].

Breeding site selection has mostly been studied at a macro environmental level. For instance, it has been shown that many species exhibit preferences of where to breed [3,4] with respect to vegetation (e.g. [5]) and water availability (e.g. [6]), habitat composition, predators, or conspecific and parasite densities (e.g. [7–9]). However, in many cases, the choice of the breeding site can be influenced by even more fine-scale parameters [3,10], such as microhabitat composition, local topography, or edge effects. For example, in two sympatric species of polar seals it was found that both use drifting ice to breed; while one species was less choosy about the specific breeding spot, the other one was highly selective about the particular topographical characteristics of the breeding site [11]. Moreover, in some species of tree frogs (Hylidae) water depth, distance to the water, temperature, and waterbody size play a key role when choosing their breeding sites [12]. The analysis of factors across different scales therefore can provide information about selective pressures that have led to differences in reproductive behaviours across and within species.

In many amphibian species, it has been shown that oviposition site selection is key for offspring survival and development (e.g. [5,7,9,13–16]). As most amphibians are oviparous and deposit their eggs directly into the environment, desiccation is one of the biggest threats to egg survival [17], especially in species that do not use permanent sources of water for egg deposition [5,18–21]. As amphibian eggs lack a protective shell [22], female frogs are expected to select oviposition locations that ensure a constant source of hydration [6,13,18,23], even when reproductive seasons are aligned with rainy seasons, as hydration levels can fluctuate [24].

Glassfrogs (family Centrolenidae) are a good model for investigating the factors that shape oviposition site selection. These Neotropical frogs deposit their clutches terrestrially [25] and many species exhibit high selectivity in their breeding sites. They mainly deposit eggs on preferred locations in the vegetation or other substrates, such as moss [26], rocks in the splash area of waterfalls, the tip of leaves, or the upper or under side of leaves [27]. Several species exhibit male or female uniparental care, which is expressed in the form of offspring guarding, hydration by brooding and defence of embryos against predators [21,28–31]. Some species have evolved extended forms of care, where parents remain and care for their clutches for almost until hatching [30]. However, many glassfrog species did not evolve such prolonged forms of care [28,30]. Those species without prolonged forms of care either leave the oviposition site directly after mating [26], or perform short-term brooding of the clutch for only a few hours after egg deposition [27,30]. While the presence of a parent may help mitigate various adverse effects at suboptimal oviposition locations, in the absence of prolonged parental care, site selection becomes even more important, as the embryos cannot escape unfavourable conditions. Therefore, the selection of a suitable oviposition location in species that lack parental care may secure constant external source of hydration, thus increasing chance of offspring survival and optimal development until hatching.

We investigated the possible benefits of selected oviposition sites within leaves in *Teratohyla spinosa* (Spiny Cochran frog)*.* This species lays the eggs on the underside of leaves close to their margins and has short-term maternal care [27]. We hypothesised that the close contact of the clutches with the margins of the leaves offer direct benefits to the eggs in terms of hydration, leading to enhanced development and hatching success. In the rainforest, water in the form of airborne humidity and rain usually contacts the surface of vegetation before evaporating or falling to the ground. When leaf surfaces are saturated with water, fused drops will slide from the edges to the tip of the leaves before dripping (AVA, FNAL, JC pers. obs.). Therefore, we expected that eggs deposited on leaf margins would experience increased hydration as water flows over them. We experimentally tested whether the position of the clutch on the leaf affects its hydration level, embryo development and embryo survival.

## Materials and methods

### Study site

**Fig 1:**
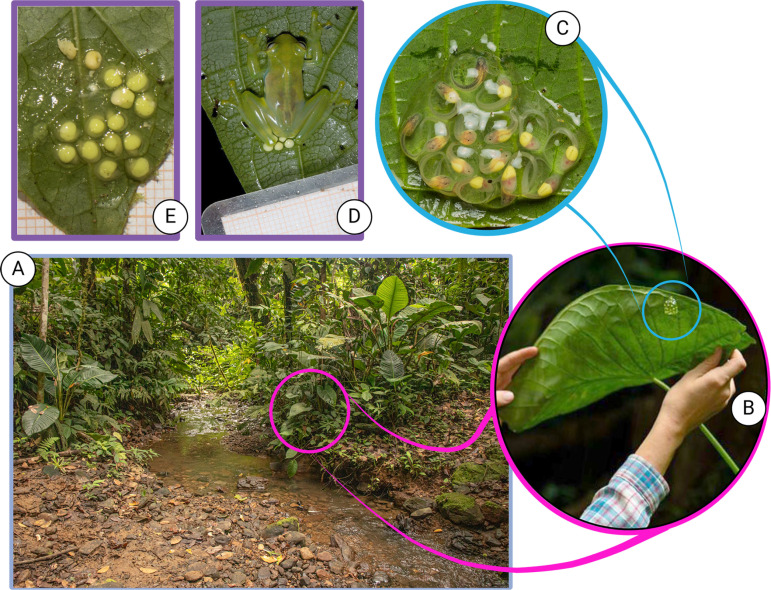
Study system. A) Sampling was done throughout 500 m of a creek surrounded by secondary forest, B) Clutches of *Teratohyla spinosa* are deposited on the underside of leaves near the margins, C) Detail of an old clutch, D) Mother of *T. spinosa* performing brooding after oviposition, E) Clutch of *T. spinosa* deceased by desiccation. Photos A by FNAL and B-E by JC. Created with BioRender.com.

Our study took place in Canandé Reserve, Esmeraldas province, Ecuador, along a 500 m length of a creek (0° 31' 24.7'' N, 79° 12' 45.6'' W). The reserve is located between two Biodiversity hotspots: Tumbes-Chocó-Magdalena and Tropical Andes [32], within the Lowland Evergreen Forest ecosystem [33]. The site is characterized by tropical, humid, seasonal weather and a rainy season that takes place from November to May. The creek floor and margins are heterogeneous, changing from flat boulders to clay. The vegetation corresponds to secondary forest, with old canopy trees higher than 10 m and herbaceous plants densely covering the margins of the creek (Fig. 1). The transect was monitored daily.

### Study species

**Fig 2:**
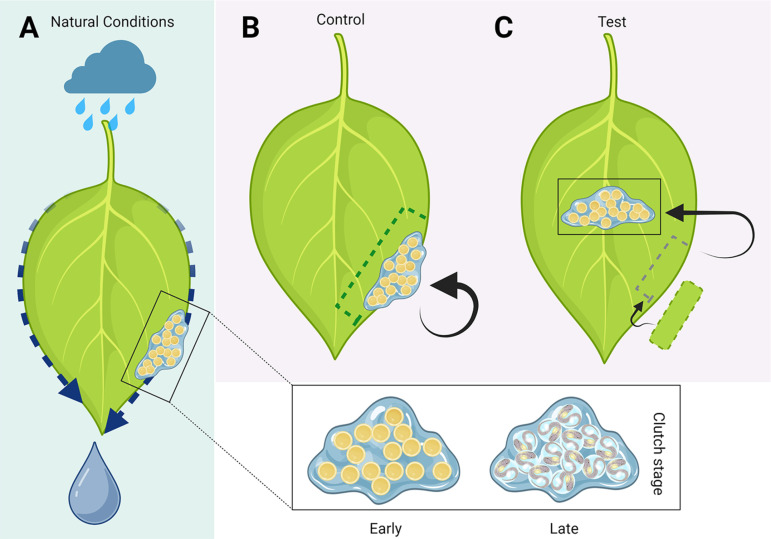
Experimental design to test the effect of clutch position on embryonic survivorship. A) Natural conditions, with the egg clutch located on the edge of the underside of a leaf. The box presents two example illustrations of clutches at an early versus late developmental stage (see Methods). B) Control clutches: placed at the edges of leaves. C) Experimental clutches: relocated to the centre of the leaf, avoiding contact with the edges. The hole left by the cut area was patched by sewing a piece of a different leaf from the same plant. Created with BioRender.com.

*Teratohyla spinosa* (Centrolenidae) is a small (<2.5 cm), nocturnal glassfrog, found in the vegetation alongside rivers, small streams, and creeks [27]. The reproductive activity of *T. spinosa* occurs mostly during the rainy season, when males vocalize mainly on the upper side of leaves to attract mates. Females approach males to engage in amplexus for several hours. During this time, the amplectant pair will move across the vegetation in the close surroundings until selecting a leaf. The female will deposit a jelly-rich clutch of eggs on the underside of the selected leaf, close to the edges (Fig 2A, [27]). Neither males nor females of *T. spinosa* show prolonged parental care, and leave the oviposition site soon after mating, but females remain for a few hours brooding the clutch before leaving [34]. We had previously observed desiccated clutches of this species in the experimental site (FNAL, JC, LJ pers. obs).

### Field work – experiment

The experiment was carried out from April 16^th^ to June 11^th^, 2022. Clutches were randomly assigned to the experimental or control condition. In the experimental condition, we cut out the leaf area surrounding the clutch (5-10 mm away from the clutch margin) to avoid forcibly removing the clutch and sewed it with thread and needle in the centre of the leaf, aiming for the point at maximal distance from all leaf edges. In the control condition, we cut out the clutch in the same way as the experimental condition, but we sewed the clutch back to the original location, to ensure similar handling procedures of all clutches across conditions (Fig 2B). To increase stability of all leaf patches, we additionally sewed another piece of leaf from the same plant on top of the reattached piece of test and control clutches. To maintain the original shape of all leaves in the test condition, we patched a piece of leave to the removed area (Fig 2C). This would allow for normal water droplet movement along the edges.

### Data collection

For all clutches we assessed level of hydration, which was measured by the thickness (mm) of the clutch at the highest point, indicated generally by the biggest live embryos/live embryos clumped together. We measured thickness using lateral views of the clutches measuring from the highest point of the clutch to the nearest leaf point in a 90° angle. This method is an adaptation from the method used by [28] where thickness is measured with a probe inserted into the clutch. We did not use this method to avoid premature hatching of the embryos in late stages due to repeated manipulations for those measurements. We took zenithal photographs to measure clutch area (mm^2^), with the outer limit of a clutch defined by the jelly margin. We also counted the number of eggs alive and dead/unfertilized in each clutch on each observation. Embryos were classified as not alive if they exhibited signs of decomposition and/or changes in coloration, or remained in the same body position and developmental stage for several days. We noted the age of each clutch, measured as days since oviposition. Oviposition time was estimated based on developmental stage of the embryos or we set oviposition time “zero” for clutches found the next day after observing amplexus. We recorded the stage of development of the residing embryos, dividing this into *early* stages (from oviposition to stage 17 [35,36]) and *late* stages (from stage 18 [35,36] until hatching). We also recorded the hatching period, referring specifically to the time it took the clutch to fully hatch, counting the days from the hatching of the first tadpole until the last one. We calculated the ratio between the area of the clutch divided by the number of eggs it possessed. Additionally, we measured the width of the leaf (cm) at its thickest point. We obtained daily temperature and humidity data provided to us from a nearby weather station [37], located within 2 km from our study site. We calculated the Temperature and Humidity Index (THI) with the formula THI =  0.8 * T +  RH * (T-14.4) +  46.4, following [38] using the average daily values. Since hatching is not synchronous for all embryos, and glassfrog species may hatch prematurely [39], we also recorded the number of days until the clutch reached hatching capacity of the larvae, defined as the stage at which larvae can survive even when hatching prematurely [39]. Additional variables we recorded were the number of days spent in the experiment and percentage of mortality, defined as the percentage of alive vs dead embryos inside the clutch prior to complete hatching.

All clutches were monitored twice a day, once during the day and once at night, until all tadpoles of the clutch had hatched or died. We considered mortality as death by desiccation, predation, fungal infection, parasitism, lack of fertilization, or unknown causes. During each observation we turned over the leaves and took lateral and zenithal photographs of the clutch with measuring paper. All photos were analysed using ImageJ (version 1.53r).

Our study followed Good Scientific Practice (GSP) guidelines and the ASAB for the ethical treatment of nonhuman animals in behavioural research and teaching [40]. The Jocotoco Research Station and the ‘Ministerio del Ambiente, Agua y Transición Ecológica’ (permit number: MAATE-DBI-CM-2022-0245) granted us working permits.

### Statistical analyses

We conducted all statistical analyses in R v3.6.0 (R Core Team 2020) using RStudio (RStudio Team 2020). We report our results following [41]. We used a Bayesian framework to determine what factors influence the thickness, mortality, and development rate of a clutch. We assumed statistical significance if the 95% credible intervals did not overlap 0.

We used a Gaussian Bayesian linear mixed model (MCMCglmm package, [42]) to determine the effect of clutch position on hydration with thickness as the response variable. As fixed effects, we added an interaction between the condition (relocated *versus* control) and the days since oviposition, as well as the width of the leaf, the daily THI, and the number of eggs per area. To account for the repeated measures, we also added the clutch ID as a random effect. We used a weak prior for one response and one random variable (see Supplementary Material S1).

We built a Bayesian model with a Poisson distribution (MCMCglmm package, [42]) to determine what factors influence hatching time. As a response variable, we used hatching period, and as fixed effects we used condition, the average THI, and the average number of eggs per area. Because hatching period was a non-integer, we multiplied the values by ten to allow the model to run. We used a weak prior for one response variable (see Supplementary Material S1).

For these two models, we set a number of iterations of 1 000 000, with a burnin of 10 000 and a thinning interval of 100. We verified the absence of autocorrelation (correlation between lags <  0.1), that sufficient mixing was reached (plots of MCMC chains), and that we ran the Markov chain long enough (Heidelberg and Welch diagnostic tests; [42]).

To determine what factors influence the mortality rate of a clutch, we built a zero-inflated beta regression in a Bayesian framework (brms package, [43]). We set the mean, phi and zero-inflated part similarly, with the percentage of mortality at the end of the experiment as a response variable, and as fixed effects the condition, the average THI, the average number of eggs per area and the average thickness of the clutch. Using the “get_prior” function, we set a specific prior (3, 0, 2.5) for the intercept class elements and a normal prior for the b class elements. We ran this model 2 000 times on 4 chains with a warmup of 1 000. Because beta regression does not allow for ones, we set all ones to 0.999 to allow the model to run. We verified the absence of autocorrelation and sufficient mixing using diagnostic plots.

Additionally, we performed a univariate COX (proportional-hazards) regression [44] to obtain survival probability differences between conditions with position on the leaf as hazard risk and days since oviposition as the response variable. In this model we considered the clutch as a unit and did not account for death events of individual embryos, meaning we calculated the survival probability of clutches rather than embryos. Censored clutches, i.e., clutches for which information of death (at any point in the life of the individuals within the clutch) is missing or incomplete, included clutches with more than 50% embryos hatched or clutches that did not have hatched embryos until the end of the experiment but were still alive. We assumed statistical significance if the 95% credible confidence intervals did not overlap 1.

## Results

Throughout the experimental period we found 41 clutches, all deposited at the margins of leaves. Of those, 17 were assigned to the experimental (centre) and 24 to the control (edge) condition. Clutches were found across the creek at height ranging between 1 to 5 m, and up to 1 to 2 m from the water’s edge into the forest. Successful hatching of 100% of embryos occurred in 15 clutches (5 =  experimental; 10 =  control) while 100% mortality occurred in 10 clutches (5 =  experimental; 5 =  control). On average, the clutches we found had 19 (SD =  3.29) embryos per clutch. Clutches took on average 8.16 (SD = 0.66) days until they reached hatching capacity. As hatching is asynchronous, we calculated the time it took for all tadpoles of a clutch to successfully hatch. We found that it took on average 11.62 (SD = 1.99) days after oviposition until the first tadpole hatched, and approximately another 3.75 (SD = 1.96) days until hatching period was completed.

**Fig 3:**
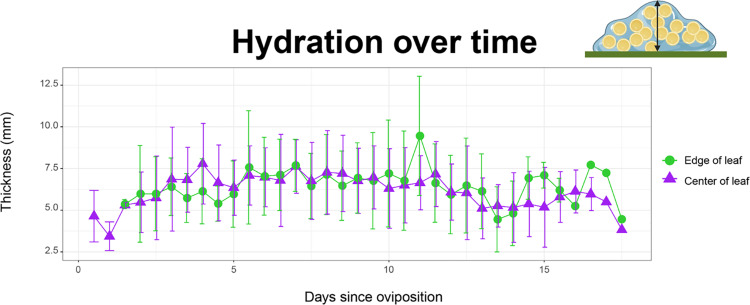
Hydration levels in control clutches (placed at the edges of leaves) and experimental clutches (relocated to the centre of the leaf) of *Teratohyla spinosa.* Average clutch thickness per day for both experimental conditions: clutches on the edge (control) and on the centre of the leaves (translocated). Layout created with BioRender.com.

We did not find clear evidence that the condition, the days since oviposition, the width of the leaf, or the daily THI, were associated with the level of hydration of a clutch (*p*MCMC >  0.05, Table 1; Fig 3). There was weak indication that with more eggs per area the level of hydration increased, but results were only marginally significant (Table 1; 95% CI =  [-19.90; 0.079], *p* =  0.053).

**Table 1:** - Summary of confidence intervals and *p* value of MCMC model for level of hydration of clutch.

	Lower 95% CI	Upper 95% CI	*p*MCMC
Intercept	0.209	16.299	0.057
Condition	-1.509	1.2151	0.853
Days since oviposition	-0.130	0.010	0.100
Width of leaf	-0.020	0.056	0.336
THI	-0.112	0.103	0.914
Number of eggs per area	-19.909	0.079	0.053
Condition*days since oviposition	-0.143	0.033	0.218

We also found no evidence that the condition, the average THI, the average number of eggs per area, or the average thickness of the clutch were associated with mortality rate of the clutch (N =  7/17 with more than 50% mortality in control clutches, N =  7/24 with more than 50% mortality in experimental clutches; Table 2).

**Table 2:** - Summary of estimates and confidence intervals for zero-inflated beta regression model. S.E.: Standard Error; CI: Confidence Interval.

	Estimate	S.E	Lower 95% CI	Upper 95% CI
Intercept	12.79	32.37	-48.85	77.02
Φ Intercept	11.12	29.92	-47.67	71.51
Zi Intercept	19.6	20.21	-6.95	66.14
Condition	-0.48	0.58	-1.62	0.65
THI	-0.15	0.44	-1.02	0.69
Number of eggs per area	-0.09	1.03	-2.11	1.92
Thickness	-0.15	0.22	-0.6	0.28
Φ Condition	0.03	0.57	-1.09	1.16
Φ THI	-0.17	0.4	-0.98	0.63
Φ Number of eggs per area	-5.07	11.54	-27.49	17.25
Φ Thickness	0.06	0.24	-0.39	0.55
Zi Condition	0.84	0.83	-0.69	2.56
Zi THI	-0.34	0.28	-0.98	0.01
Zi Number of eggs per area	25.99	21.38	-13.53	69.3
zi Thickness	0.43	0.28	-0.1	0.99

**Fig 4:**
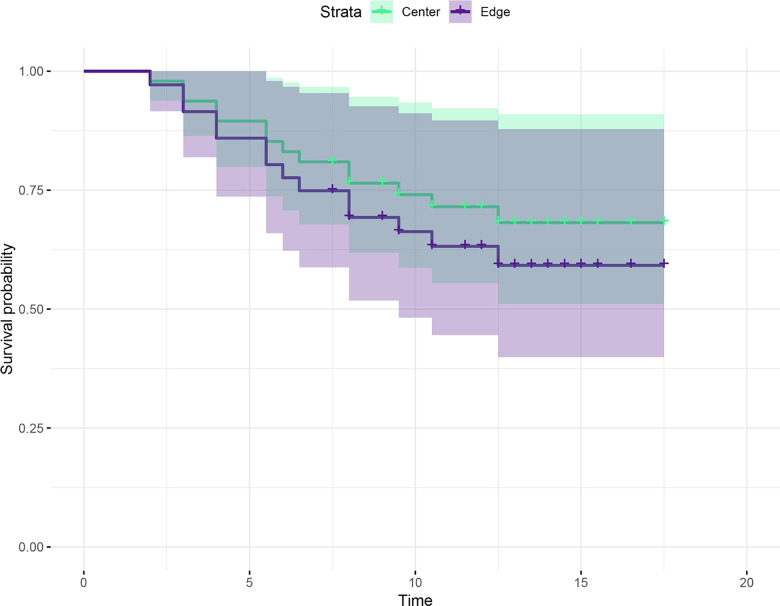
Survival probability of control clutches (placed at the edges of leaves) and experimental clutches (relocated to the centre of the leaf) of *Teratohyla spinosa.* Probability of survival in both conditions decreased similarly with time (*p* =  0.6).

From the univariate COX regression, we found weak evidence for differences in clutch survival between conditions. The probability of death was 27% higher in experimental compared to control clutches, however, this result was not statistically significant (Fig.4, HR =  0.73, 95%; CI =  [0.48; 3.91], *p* =  0.6).

Lastly, we found no evidence that the condition, THI or eggs per area influenced the number of days it took until full hatching (Table 3).

**Table 3:** Summary of confidence intervals and *p* MCMC model for time of hatch.

	Lower 95% CI	Upper 95% CI	*p*MCMC
Intercept	-15.56	74.28	0.4183
Condition (centre-edge)	-0.91	0.49	0.575
THI	-0.97	0.25	0.227
Eggs per area	-6.82	29.98	0.206

## Discussion

Contrary to our expectations, we found that clutch hydration and mortality were not affected by the location on the leaf. Test and control clutches did not differ in thickness throughout their development. This suggests that hydration is not improved in clutches located at the edge of the leaf and that there might be different reasons that have led to the evolution of this oviposition pattern in this species. This finding was not what we had expected, given the big threat that desiccation poses to terrestrial/arboreal clutches [45]. In some glassfrog species, parents remain with their eggs almost throughout the entire development period and actively hydrate their eggs when sitting on top of them (e.g., *Centrolene peristicta* [46], *Hyalinobatrachium aureoguttatum* [16], *Ikakogi tayrona* [47]). Parental removal experiments have shown that the absence of the caring parent leads to a considerable increase in offspring mortality rates due to dehydration or predation [46,48–50]. However, many species of glassfrogs do not exhibit extended parental care and only perform brood once just after oviposition to boost hydration in the jelly-rich clutches [29,30]; although, it is not the case for *Espadarana prosoblepon* [26]. *Teratohyla spinosa* eggs, along with those of other species that lack extended parental care, are embedded in a highly absorbent jelly matrix. It has been proposed for these species that the jelly may be fit for withstanding desiccation without the need for active hydration by the parents under controlled, semi-natural conditions ([34], although in this experiment sample size for *T. spinosa* was N =  1). This is similar to what happens in other amphibian species that produce froth nests [51].

In our study, we found that under field conditions the embryos do not show signs of dehydration likely due to humidity levels being constantly high and making desiccation risk very low during the rainy season. Hydric stress has proven to be a driver for changes in developmental rate and hatching plasticity in other species [19,39,52]. We did not observe any signs of hydric stress during development in our study, as test and control clutches did not differ in hatching times. Likewise, there was no indication of hydric stress affecting survivorship, as mortality rates did not differ between clutch position on leaves. The absence of significant differences in our dataset does not automatically imply that hydration did not play any role in the evolution of the specific oviposition pattern in our study species. One possible reason for this lack of difference could be that, although we did not measure the weather conditions per oviposition site, and there were daily and periodically considerable shifts in temperature and humidity (FNAL, AVA, JC, JGTR, LJ, MGP pers. obs.), these were not extreme enough to impact the clutches during the entire experiment, as they were performed during the peak of the rainy season [53]. These conditions might change especially at the beginning and the end of the rainy season when humidity is lower, and rainfall is scarcer [54]. Eventually, clutches may benefit from increased hydration during periods of low rainfall [55]. Moreover, highly unpredictable weather conditions are more frequent due to climate change, making amphibians more susceptible to extreme weather shifts [18], especially those with terrestrial reproduction [21]. Therefore, future studies should investigate the effects of more variable environmental conditions on clutch development and survival.

Alternatively, there could also have been other factors that might have shaped this specific oviposition pattern in our study species. Refsnider and Janzen [1] summarized drivers for oviposition site selection into six categories: 1) maximizing embryo survival, 2) maximizing maternal survival, 3) modifying offspring phenotype, 4) proximity to suitable habitat for offspring, 5) maintaining natal philopatry, and 6) indirect oviposition-site choice via mate choice. In our study, we observed no differences to offspring survival or hatching time between clutch position on leaves. Regarding indirect site-selection via mate choice, we observed males calling from the same areas but different leaves on consecutive nights, suggesting some form of site fidelity. Additionally, we observed that males and females moved over several leaves during mating. This might indicate active participation by both sexes in oviposition site selection. By choosing the most suitable mate, the female simultaneously selects an oviposition site on a macro scale. However, the decision of the exact location to deposit the eggs at a micro-scale remains unclear. On one hand, there might be benefits for the females associated to the egg laying process itself (i.e., it might be easier for them to lay eggs on the edges of leaves). On the other hand, there might also be benefits to the offspring such as enhanced hatching success, or benefits in a predation context [56]. These hypotheses remain highly speculative as we did not include such factors in our analysis, but should be considered in future studies.

In summary, we found no evidence that clutch placement on the edge of leaves matters for embryonic development and hatching success. Eventually, an effect might only become apparent at later developmental stages or under more extreme climatic conditions. Alternatively, the placement of clutches at the edges of leaves might reflect some benefit to the female during the egg laying process. Future studies should therefore test for further possible adaptive benefits of particular clutch placement across different species.

## Supporting information

S1 Text Priors used in analyses. (DOCX)

S2 Text Spanish version of manuscript. (DOCX)
