# Supplementary material for: Is there a right place? The effect of within-leaf clutch location on offspring survival in a glassfrog
Source: PLoS One. 2025 Apr 11;20(4):e0309642. doi: 10.1371/journal.pone.0309642 (PMC11990747; doi:10.1371/journal.pone.0309642)
Supplement: S1 Text — (DOCX) [file pone.0309642.s001.docx]

**S1.- Priors used in statistical analyses**

***Prior 1.-*** Code used in MCMCglmm model of hydration level.

# weak prior for fixed (R) and random effects (G)

prior1 <- list(R = list(V = 1, nu = 0.002), G=list(G1=list(V=1, nu=0.002)))

***Prior 2.-*** Code used for brm function on mortality analysis. Priors modified from default priors.

See full code in repository.

priors <- c(set_prior("student_t(3, 0, 2.5)", class = "Intercept"),

set_prior("normal(0, 1)", class = "b"))

***Prior 3.-*** Code used in MCMCglmm model of developmental rate (hatching time).

# weak prior for fixed (R) effect dev rate

prior2 <- list(R = list(V = 1, nu = 0.002))
