# Supplementary material for: Is there a right place? The effect of within-leaf clutch location on offspring survival in a glassfrog
Source: PLoS One. 2025 Apr 11;20(4):e0309642. doi: 10.1371/journal.pone.0309642 (PMC11990747; doi:10.1371/journal.pone.0309642)
Supplement: S2 Text — (DOCX) [file pone.0309642.s002.docx]

**S1.- Translation of text to Spanish**

**¿Existe un lugar adecuado? El efecto de la selección del sitio de oviposición dentro de la hoja en la supervivencia de la descendencia en una rana de cristal**

Francesca N. Angiolani-Larrea^1^, Anyelet Valencia-Aguilar^1,2^, Marina Garrido-Priego^1^, Mélissa Peignier ^1^, Jaime Culebras^3,4^, Lelis Jindiachi^1,5^, José G. Tinajero-Romero^1,6^, Juan M. Guayasamin^7,8^, Eva Ringler^1^.

^1^Division of Behavioural Ecology, Institute of Ecology and Evolution, University of Bern, Bern, Switzerland

^2^ Institute of Animal Physiology, Department of Animal Physiology and Molecular Biomedicine, Justus-Liebig-University Giessen, Giessen, Germany

3 Photo Wildlife Tours, Quito, Ecuador

4Fundación Cóndor Andino, Quito, Ecuador

^5^Pueblo Shuar Arutam, Federación Interprovincial Centros Shuar (FICSH), Sucúa, Ecuador

^6^ Escuela de Biologia, Universidad de Costa Rica, San José, Costa Rica

^7^ Universidad San Francisco de Quito USFQ, Laboratorio de Biología Evolutiva, Colegio de Ciencias Biológicas y Ambientales COCIBA, Instituto BIÓSFERA-USFQ, Campus Cumbayá, Quito 170901, Ecuador.

^8^ Tandayapa Cloud Forest Station, Universidad San Francisco de Quito USFQ, P.O. Box 17-1200-841, Quito, Ecuador.

**Resumen**

Saber escoger el lugar para criar puede tener consecuencias fundamentales para el desarrollo y la supervivencia de la descendencia. En los anfibios, la desecación es una de las mayores amenazas para la supervivencia de la descendencia, especialmente en las especies que depositan sus puestas en hábitats terrestres. En varias especies, la hidratación de la puesta está asegurada por un progenitor que cuida, pero en las especies sin cuidados prolongados, la selección del sitio se vuelve extremadamente importante para asegurar fuentes externas constantes de hidratación. Utilizamos la Rana de cristal espinosa (*Teratohyla spinosa*), una rana de cristal neotropical en la que las hembras realizan sólo una hidratación de corto plazo de las puestas para después ambos progenitores abandonar a la descendencia, para probar el efecto de la selección del sitio de oviposición dentro de las hojas en el desarrollo y la supervivencia de la descendencia. Observaciones anteriores han revelado que esta especie prefiere depositar los huevos en el envés de las hojas cerca de sus márgenes. Planteamos la hipótesis de que *T. spinosa* elige estratégicamente este lugar para asegurar la hidratación de la puesta durante el desarrollo embrionario, ya que las gotas de agua se deslizan por los márgenes hasta la punta de las hojas antes de gotear. Para ello, realizamos un experimento de translocación de puestas en el que manipulamos la ubicación de las puestas colocándolas lejos del margen de las hojas y comparamos su nivel de hidratación, tiempo de eclosión y tasa de mortalidad con las puestas que se mantuvieron en los márgenes de las hojas. Contrariamente a nuestras expectativas, descubrimos que la hidratación y la mortalidad de las puestas no se vieron afectadas por la ubicación en la hoja. Estos hallazgos sugieren que cuando esta especie deposita las puestas en los márgenes de la hojas no se observan mejoras en las condiciones de hidratación, al menos en condiciones de alta humedad.

**Introducción**

En muchas especies animales, la elección del lugar de crianza puede tener un fuerte impacto en la supervivencia y desarrollo de las crías [1,2]. Estas decisiones no suelen ser aleatorias y están influidas por factores como la competencia intraespecífica, la estructura del hábitat, la presión de la depredación y las variables climáticas [2]. Por lo tanto, se espera que los individuos equilibren entre los recursos que invierten para su descendencia actual o futura y sus propias necesidades [1].

La selección del sitio de crianza se ha estudiado principalmente a nivel macroambiental. Por ejemplo, se ha demostrado que muchas especies muestran preferencias de dónde criar [3,4] con respecto a la vegetación (p. ej.: [5]) y la disponibilidad de agua (p. ej.: [6]), la composición del hábitat, los depredadores o las densidades de congéneres y parásitos (p. ej.: [7–9]). Sin embargo, en muchos casos, la elección del sitio de crianza puede estar influenciada por parámetros de escala aún más fina [3,10], como la composición del microhábitat, la topografía local o los efectos de borde. Por ejemplo, en dos especies simpátricas de focas polares se encontró que ambas utilizan hielo a la deriva para reproducirse; mientras que una especie era menos exigente con el sitio de crianza específico, la otra era altamente selectiva con respecto a las características particulares de la topografía del sitio de crianza [11]. Además, en algunas especies de ranas arbóreas (Hylidae) la profundidad del agua, la distancia al agua, la temperatura y el tamaño del cuerpo de agua juegan un papel clave al elegir sus sitios de crianza [12]. Por lo tanto, el análisis de los factores a diferentes escalas puede brindar información sobre las presiones selectivas que han llevado a diferencias en los comportamientos reproductivos tanto entre especies como dentro de ellas.

En muchas especies de anfibios, se ha observado que la selección del sitio de oviposición es clave para la supervivencia y el desarrollo de la descendencia (p. ej.: [5,7,9,13–16]). Como la mayoría de los anfibios son ovíparos y depositan sus huevos directamente en el ambiente, la desecación es una de las mayores amenazas para la supervivencia de los huevos [17], especialmente en especies que no utilizan fuentes permanentes de agua para depositar los huevos [5,18–21]. Como los huevos de anfibios carecen de una cáscara protectora [22], se espera que las hembras de las ranas seleccionen lugares de oviposición que garanticen una fuente constante de hidratación [6,13,18,23], incluso cuando las temporadas reproductivas son durante las temporadas de lluvia, ya que los niveles de hidratación pueden fluctuar [24].

Las ranas de cristal (familia Centrolenidae) son un buen modelo para investigar los factores que determinan la selección del sitio de oviposición. Estas ranas neotropicales depositan sus puestas fuera del agua [25] y muchas especies muestran ser muy selectivas para escoger el sitio donde criar a su descendencia. Depositan principalmente los huevos en lugares específicos en la vegetación u otros sustratos, como el musgo [26], rocas en el área de salpicadura de las cascadas, la punta de las hojas o el haz o envés de las hojas [27]. Varias especies muestran un cuidado uniparental masculino o femenino, que se manifiesta mediante vigilancia de las crías, hidratación colocándose sobre los embriones y defensa contra depredadores [21,28–31]. Algunas especies han desarrollado formas prolongadas de cuidado, donde los padres permanecen y cuidan a sus puestas casi hasta la eclosión [30]. Sin embargo, muchas especies de ranas de cristal no desarrollaron estas formas tan prolongadas de cuidado [28,30]. Las especies sin cuidado prolongado abandonan el lugar de oviposición justo después del apareamiento [26] o realizan un cuidado de la puesta a corto plazo sólo durante unas horas después de la puesta de huevos [27,30]. Si bien la presencia de un progenitor puede ayudar a mitigar varios efectos adversos en lugares de oviposición no tan óptimos, en ausencia de cuidados parentales prolongados, la selección del sitio se vuelve aún más importante, ya que los embriones no pueden escapar de condiciones desfavorables. Por lo tanto, la selección de un lugar de oviposición adecuado en especies que carecen de cuidados parentales puede asegurar una fuente externa constante de hidratación, aumentando así la posibilidad de supervivencia de la descendencia y un desarrollo óptimo hasta la eclosión.

Investigamos los posibles beneficios de los sitios de oviposición seleccionados en las hojas en *Teratohyla spinosa* (Rana de cristal espinosa). Esta especie pone los huevos en el envés de las hojas cerca de sus márgenes y tiene cuidados maternales a corto plazo [27]. Planteamos la hipótesis de que el contacto cercano de las puestas con los márgenes de las hojas ofrece beneficios directos a los huevos por hidratación, lo que conduce a un mejor desarrollo y éxito de eclosión. En la selva tropical, el agua presente en la humedad atmosférica y lluvia suele entrar en contacto con la superficie de la vegetación antes de evaporarse o caer al suelo. Cuando la superficie de las hojas está saturada de agua, las gotas fusionadas se deslizarán desde los márgenes hasta la punta de las hojas antes de gotear (AVA, FNAL, JC, obs. Pers.). Por lo tanto, esperábamos que los huevos depositados en los márgenes de las hojas experimentaran una mayor hidratación debido a que el agua fluye sobre ellos. Probamos experimentalmente si la posición de la puesta en la hoja afecta su nivel de hidratación, el desarrollo embrionario y la supervivencia del embrión.

**MATERIALES Y MÉTODOS**

**Sitio de estudio**

Nuestro estudio se llevó a cabo en la Reserva Canandé, provincia de Esmeraldas, Ecuador, a lo largo de 500 m de longitud de un arroyo (0° 31' 24.7'' N, 79° 12' 45.6'' O). La reserva está ubicada entre dos Hotspots de biodiversidad: Tumbes-Chocó-Magdalena y Andes tropicales [32], y su ecosistema es el Bosque Siempreverde de Tierras Bajas [33]. El sitio se caracteriza por un clima tropical, húmedo y estacional y una temporada de lluvias que tiene lugar de noviembre a mayo. El lecho y orillas del arroyo son heterogéneos, variando desde cantos rodados planos a arcilla. La vegetación corresponde a bosque secundario, con árboles viejos de dosel superior a 10 m y plantas herbáceas que cubren densamente las orillas del arroyo (Fig. 1). El transecto fue monitoreado diariamente.


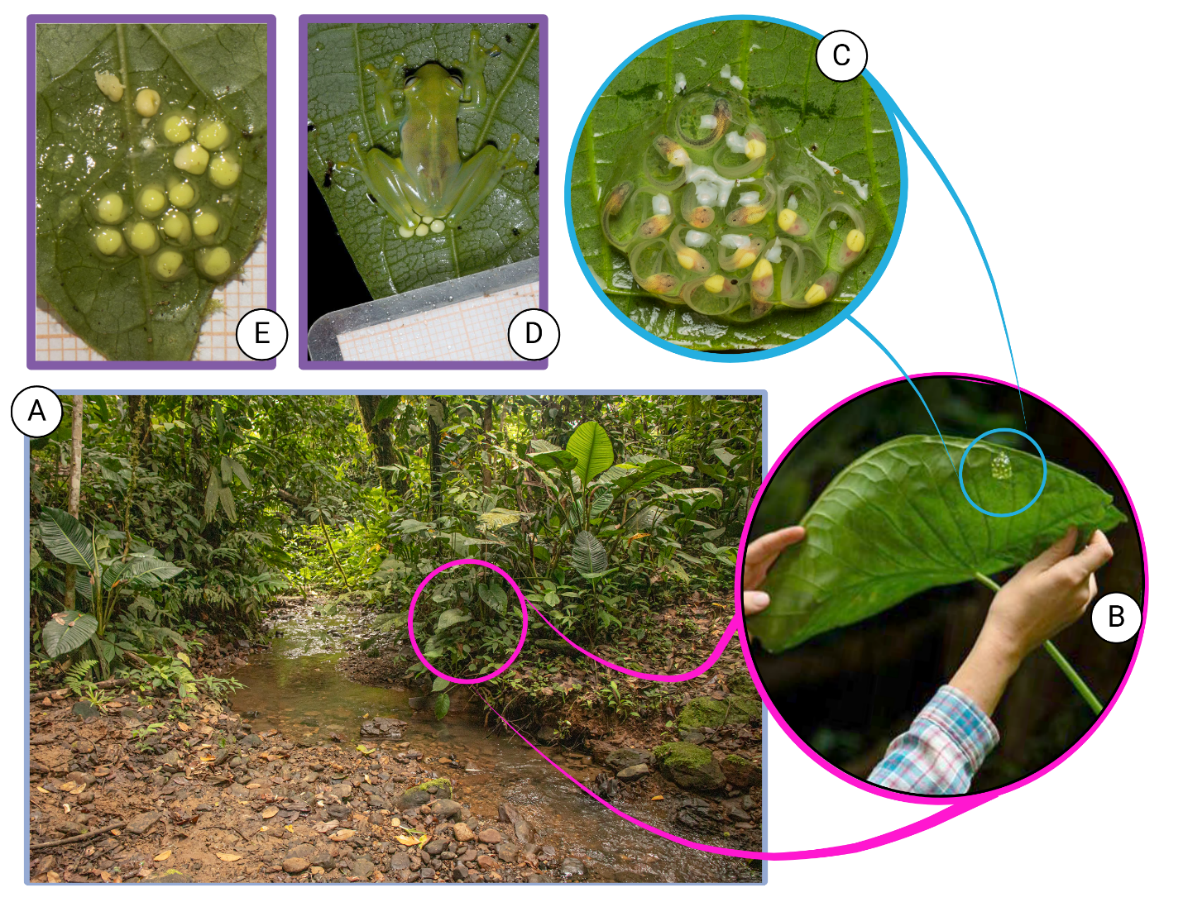


**Figura 1.-** Sistema de estudio. A) El muestreo se realizó a lo largo de 500 m de un arroyo rodeado de bosque secundario, B) Las puestas de *Teratohyla spinosa* se depositan en el envés de las hojas cerca de los márgenes, C) Detalle de una puesta antigua, D) Madre de *T. spinosa* realizando la crianza después de la oviposición, E) Puesta de *T. spinosa* muerta por desecación. Fotos A por FNAL y B-E por JC. Creado con BioRender.com

**Especie de estudio**

*Teratohyla spinosa* (Centrolenidae) es una rana de cristal pequeña (<2.5 cm), de hábitos nocturnos, que se encuentra en la vegetación de ríos, arroyos y riachuelos [27]. La actividad reproductiva de *T. spinosa* ocurre especialmente durante la temporada de lluvia, cuando los machos vocalizan principalmente en el haz de las hojas para atraer a las hembras. Las hembras se acercan a los machos para participar en el amplexo durante varias horas. Durante este tiempo, la pareja amplexada se moverá por la vegetación en los alrededores cercanos hasta seleccionar una hoja. La hembra depositará una puesta de huevos rica en gelatina en el envés de la hoja seleccionada, cerca de los márgenes (Fig. 2A, [27]). Ni los machos ni las hembras de *T. spinosa* muestran un cuidado parental prolongado y abandonan el sitio de oviposición poco después del apareamiento, pero las hembras permanecen durante unas horas cuidando la puesta antes de irse [34]. Habíamos observado previamente puestas desecadas de esta especie en el lugar del experimento (FNAL, JC, LJ obs. pers.).


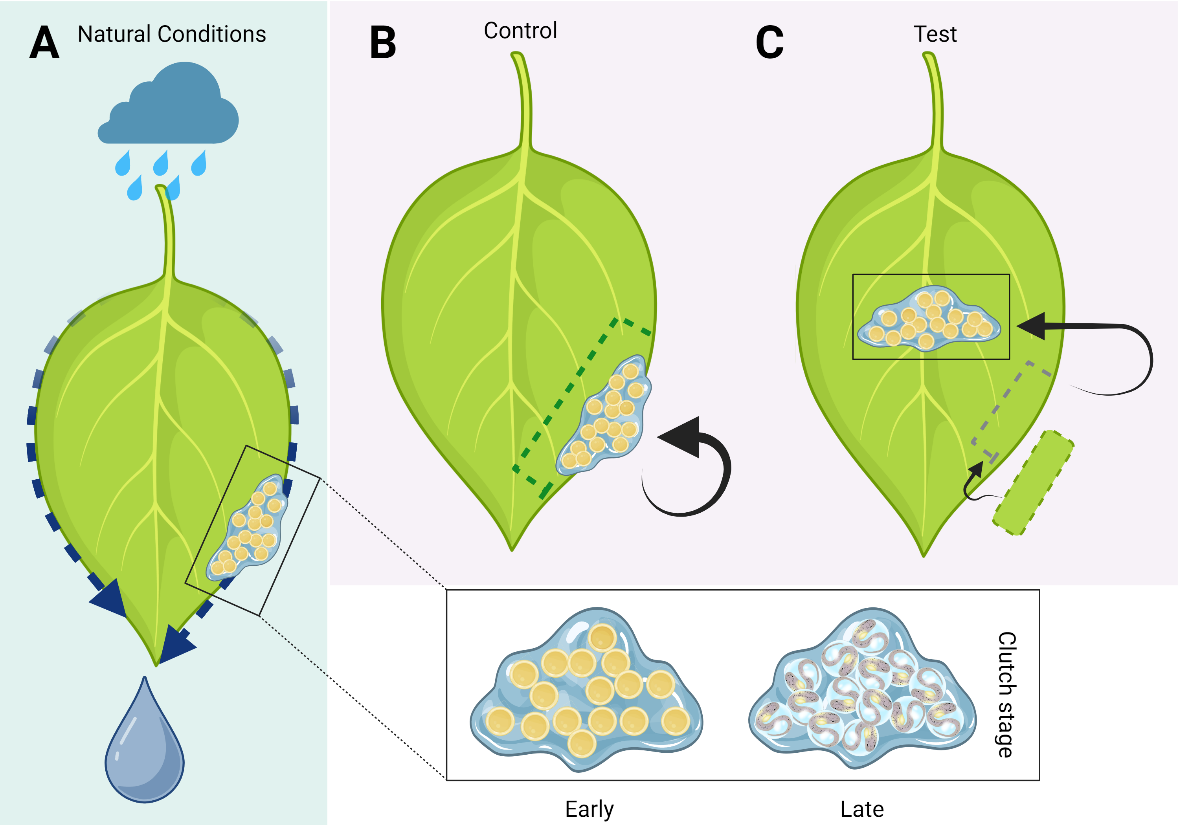


**Figura 2.-** Diseño experimental para comprobar el efecto de la posición de la puesta sobre la supervivencia embrionaria. A) Condiciones naturales, con la puesta de huevos situada en el margen del envés de una hoja. El recuadro presenta dos ejemplos de puestas en etapa temprana y tardía del desarrollo (ver Métodos). B) Puestas de control: situada en el margen de la hoja. C) Puestas experimentales: reubicada en el centro de la hoja, evitando el contacto con los márgenes. El hueco del área cortada se tapó cosiendo un trozo de una hoja diferente de la misma planta. Creado con BioRender.com

**Trabajo de campo – experimento**

El experimento se llevó a cabo del 16 de abril al 11 de junio de 2022. Las puestas se asignaron aleatoriamente a la condición de experimento o de control. En la condición de experimento, cortamos el área de la hoja que rodea la puesta (a 5-10 mm del margen de la puesta) para evitar retirarla a la fuerza y ​​la cosimos con hilo y aguja en el centro de la hoja, en el punto a la distancia máxima de todos los márgenes de la hoja. En la condición de control, cortamos la puesta de la misma manera que en la condición de experimento, pero la cosimos de nuevo en la ubicación original, para así asegurar procedimientos de manejo similares de todas las puestas en todas las condiciones (Fig. 2B). Para aumentar la estabilidad de todos los parches de hojas, adicionalmente cosimos otro trozo de hoja de la misma planta sobre el trozo reconectado de las puestas de prueba y control. Para mantener la forma original de todas las hojas en la condición de prueba, cosimos un trozo de hoja en el área removida (Fig. 2C). Esto permitiría el movimiento normal de las gotas de agua a lo largo de los márgenes.

**Recolección de datos**

Para todas las puestas evaluamos el nivel de hidratación, que se midió por el grosor (mm) de la puesta en el punto más alto, indicado generalmente por los embriones vivos más grandes/embriones vivos agrupados. Medimos el grosor utilizando vistas laterales de las puestas midiendo desde el punto más alto de la puesta hasta el punto de la hoja más cercano en un ángulo de 90°. Este método es una adaptación del método utilizado por [28] donde el grosor se mide con una sonda insertada en la puesta. No utilizamos este método para evitar la eclosión prematura de los embriones en etapas tardías debido a manipulaciones repetidas para esas mediciones. Hicimos fotografías cenitales para medir el área de la puesta (mm^2^), con el límite exterior de una puesta definido por el margen de la gelatina. También contamos el número de huevos vivos y muertos/no fertilizados en cada puesta en cada observación. Los embriones se clasificaron como no vivos si exhibieron signos de descomposición y/o cambios en la coloración, o permanecieron en la misma posición corporal y etapa de desarrollo durante varios días. Anotamos la edad de cada puesta, medida en días desde la oviposición. El tiempo de oviposición se estimó en función del estadio de desarrollo de los embriones o establecimos el tiempo de oviposición como “cero” para las puestas encontradas al día siguiente de observar el amplexus. Registramos el estadio de desarrollo de los embriones residentes, dividiéndolo en estadios tempranos (desde la oviposición hasta el estadio 17 [35, 36]) y estadios tardíos (desde el estadio 18 [35, 36] hasta la eclosión). También registramos el período de eclosión, refiriéndonos específicamente al tiempo que tardó la puesta en eclosionar por completo, contando los días desde la eclosión del primer renacuajo hasta el último. Calculamos la relación entre el área de la puesta dividida por el número de huevos que poseía. Además, medimos el ancho de la hoja (cm) en su punto más grueso. Obtuvimos datos diarios de temperatura y humedad procedente de una estación meteorológica cercana [37], ubicada a 2 km de nuestro sitio de estudio. Calculamos el Índice de Temperatura y Humedad (THI) con la fórmula THI = 0,8*T + RH*(T-14.4) + 46.4, siguiendo a [38] utilizando los valores diarios promedio. Dado que la eclosión no es sincrónica para todos los embriones y las especies de ranas de cristal pueden eclosionar prematuramente [39], también registramos el número de días hasta que la puesta alcanzó la capacidad de eclosión de las larvas, definida como la etapa en la que las larvas pueden sobrevivir incluso cuando eclosionan prematuramente [39]. Las variables adicionales que registramos fueron el número de días transcurridos en el experimento y el porcentaje de mortalidad, definido como el porcentaje de embriones vivos y muertos dentro de la puesta antes de la eclosión completa.

Todas las puestas fueron monitoreadas dos veces al día, una por el día y otra por la noche, hasta que todos los renacuajos de la puesta eclosionaron o murieron. Consideramos mortalidad como muerte por desecación, depredación, infección fúngica, parasitismo, ausencia de fertilización o causas desconocidas. Durante cada observación, volteamos las hojas y tomamos fotografías laterales y cenitales de la puesta con papel métrico. Todas las fotos fueron analizadas utilizando ImageJ (versión 1.53r).

Nuestro estudio siguió las pautas de Buenas Prácticas Científicas (BPC) y la ASAB para el tratamiento ético de animales no humanos en la investigación y enseñanza del comportamiento [40]. La Estación Científica Jocotoco y el Ministerio del Ambiente, Agua y Transición Ecológica (número de permiso: MAATE-DBI-CM-2022-0245) nos otorgaron permisos de trabajo.

**Análisis estadísticos**

Realizamos todos los análisis estadísticos en R v3.6.0 (R Core Team 2020) utilizando RStudio (RStudio Team 2020). Informamos nuestros resultados siguiendo a [41]. Utilizamos un marco Bayesiano para determinar qué factores influyen en el grosor, la mortalidad y la tasa de desarrollo de una puesta. Asumimos la significancia estadística si los intervalos de confianza del 95 % no se superponían con 0.

Utilizamos un modelo mixto lineal Bayesiano Gaussiano (paquete MCMCglmm, [42]) para determinar el efecto de la posición de la puesta sobre la hidratación con el grosor como variable de respuesta. Como efectos fijos, agregamos una interacción entre la condición (reubicada versus control) y los días desde la oviposición, así como el ancho de la hoja, el THI diario y la cantidad de huevos por área. Para tener en cuenta las medidas repetidas, también agregamos el ID de la puesta como un efecto aleatorio. Usamos un prior débil para una respuesta y una variable aleatoria (ver Material complementario S1).

Construimos un modelo Bayesiano con una distribución de Poisson (paquete MCMCglmm, [42]) para determinar qué factores influyen en el tiempo de eclosión. Como variable de respuesta, utilizamos el período de eclosión y como efectos fijos utilizamos a) la condición, b) el THI promedio y c) el número promedio de huevos por área. Debido a que el período de eclosión no era un número entero, multiplicamos los valores por diez para permitir que el modelo funcionara. Utilizamos un prior débil para una variable de respuesta (ver Material complementario S1).

Para estos dos modelos, establecimos un número de iteraciones de 1 000 000, con un *burnin* de 10 000 y un intervalo de *thinning* de 100. Verificamos la ausencia de autocorrelación (correlación entre *lags* < 0.1), que se alcanzó una mezcla suficiente (gráficos de cadenas MCMC) y que ejecutamos la cadena de Markov durante el tiempo suficiente (pruebas de diagnóstico de Heidelberg y Welch; [42]).

Para determinar qué factores influyen en la tasa de mortalidad de una puesta, construimos una regresión beta inflada de ceros en un marco bayesiano (paquete *brms*, [43]). Fijamos la media, phi y la parte inflada de cero de forma similar, con el porcentaje de mortalidad al final del experimento como variable de respuesta, y como efectos fijos la condición, el THI promedio, el número promedio de huevos por área y el grosor promedio de la puesta. Utilizando la función “*get_prior*”, fijamos un prior específico (3, 0, 2.5) para los elementos de la clase de intersección y un prior normal para los elementos de la clase b. Ejecutamos este modelo 2000 veces en 4 cadenas con un calentamiento de 1000. Debido a que la regresión beta no permite unos, fijamos todos los unos en 0.999 para permitir que el modelo se ejecute. Verificamos la ausencia de autocorrelación y la mezcla suficiente utilizando gráficos de diagnóstico.

Además, realizamos una regresión COX (riesgos proporcionales) univariada [44] para obtener diferencias de probabilidad de supervivencia entre condiciones con la posición en la hoja como riesgo y los días desde la oviposición como variable de respuesta. En este modelo, consideramos la puesta como una unidad y no tomamos en cuenta los eventos de muerte de embriones individuales, lo que significa que calculamos la probabilidad de supervivencia de las puestas en lugar de los embriones. Las puestas censuradas, es decir, puestas para las que falta o está incompleta la información de muerte (en cualquier punto de la vida de los individuos dentro de la puesta), incluyeron puestas con más del 50% de embriones eclosionados o puestas que no tuvieron embriones eclosionados hasta el final del experimento pero que aún estaban vivos. Asumimos significancia estadística si los intervalos de confianza creíbles del 95% no se superponían 1.

**Resultados**

Durante el período del experimento encontramos 41 puestas, todas depositadas en los márgenes de las hojas. De ellas, 17 fueron asignadas a la condición experimento (centro) y 24 a la condición de control (margen). Las puestas se encontraron a lo largo del arroyo a una altura que oscilaba entre 1 y 5 m, y hasta de 1 a 2 m desde el borde del agua hacia el bosque. La eclosión exitosa del 100% de los embriones ocurrió en 15 puestas (5 = experimento; 10 = control) mientras que el 100% de mortalidad ocurrió en 10 puestas (5 = experimento; 5 = control). En promedio, las puestas que encontramos tenían 19 (DE = 3.29) embriones por puesta. Las puestas tardaron en promedio 8.16 (DE = 0.66) días hasta que alcanzaron la capacidad de eclosión. Como la eclosión es asincrónica, calculamos el tiempo que tardaron todos los renacuajos de una puesta en eclosionar con éxito. Descubrimos que transcurrieron en promedio 11.62 (DE=1.99) días desde la oviposición hasta que eclosionó el primer renacuajo y aproximadamente otros 3.75 (DE=1.96) días hasta que se logró la eclosión completa de la puesta.

No encontramos evidencia clara de que la condición, los días desde la oviposición, el ancho de la hoja o el THI diario estuvieran asociados con el nivel de hidratación de una puesta (*p*MCMC > 0.05, Tabla 1; Fig 3). Hubo indicación débil de que con más huevos por área el nivel de hidratación aumentó, pero los resultados fueron solo marginalmente significativos (Tabla 1; IC del 95% = [-19.90; 0.079], *valor p* = 0,053).

**Tabla 1.-** Resumen de los intervalos de confianza y valor *p* del modelo MCMC para el nivel de hidratación de la puesta.

|  | Menor 95% IC | Mayor 95% IC | *p*MCMC |
| --- | --- | --- | --- |
| Intercepto | 0.209 | 16.299 | 0.057 |
| Condición | -1.509 | 1.2151 | 0.853 |
| Días desde la oviposición | -0.130 | 0.010 | 0.100 |
| Ancho de la hoja | -0.020 | 0.056 | 0.336 |
| THI | -0.112 | 0.103 | 0.914 |
| Número de huevos por área | -19.909 | 0.079 | 0.053 |
| Condición* días desde la oviposición | -0.143 | 0.033 | 0.218 |


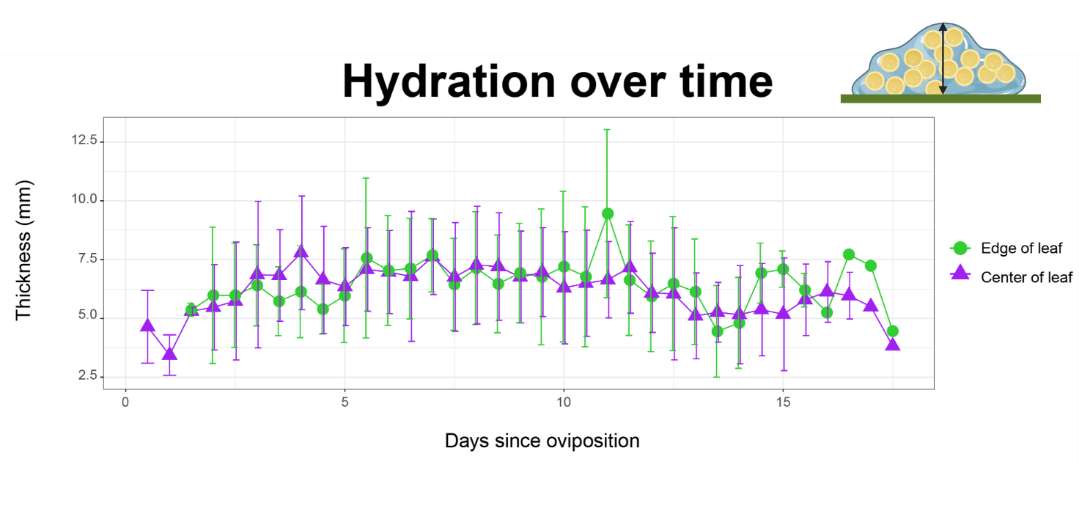


**Figura 3.-** Niveles de hidratación en las puestas control (colocadas en los márgenes de las hojas) y puestas experimentales (reubicadas en el centro de la hoja) de *Teratohyla spinosa*. Grosor medio de las puestas por día para ambas condiciones experimentales: puestas en el margen (control) y en el centro de las hojas (translocadas). Creado con BioRender.com

Tampoco encontramos evidencia de que la condición, el THI medio, el número medio de huevos por área o el grosor medio de la puesta estuvieran asociados con la tasa de mortalidad de la puesta (N = 7/17 con más del 50% de mortalidad en puestas control, N = 7/24 con más del 50% de mortalidad en puestas experimentales; Tabla 2).

**Tabla 2.-** Resumen de estimaciones e intervalos de confianza para el modelo de regresión beta inflado de ceros. E.E.: Error Estándar; IC: Intervalo de Confianza.

|  | Estimado | E.E | Menor 95% IC | Mayor 95% IC |
| --- | --- | --- | --- | --- |
| Intercepto | 12.79 | 32.37 | -48.85 | 77.02 |
| Φ Intercepto | 11.12 | 29.92 | -47.67 | 71.51 |
| Zi Intercepto | 19.6 | 20.21 | -6.95 | 66.14 |
| Condición | -0.48 | 0.58 | -1.62 | 0.65 |
| THI | -0.15 | 0.44 | -1.02 | 0.69 |
| Número de huevos por área | -0.09 | 1.03 | -2.11 | 1.92 |
| Ancho | -0.15 | 0.22 | -0.6 | 0.28 |
| Φ Condición | 0.03 | 0.57 | -1.09 | 1.16 |
| Φ THI | -0.17 | 0.4 | -0.98 | 0.63 |
| Φ Número de huevos por área | -5.07 | 11.54 | -27.49 | 17.25 |
| Φ Ancho | 0.06 | 0.24 | -0.39 | 0.55 |
| Zi Condición | 0.84 | 0.83 | -0.69 | 2.56 |
| Zi THI | -0.34 | 0.28 | -0.98 | 0.01 |
| Zi Número de huevos por área | 25.99 | 21.38 | -13.53 | 69.3 |
| zi Ancho | 0.43 | 0.28 | -0.1 | 0.99 |

A partir de la regresión de COX univariada, encontramos evidencia débil de diferencias en la supervivencia de las puestas entre las condiciones. La probabilidad de muerte fue un 27% mayor en las puestas del experimento en comparación con las del control; sin embargo, este resultado no fue estadísticamente significativo (Fig. 4, HR = 0.73, 95%; IC = [0.48; 3.91], valor *p* = 0.6).


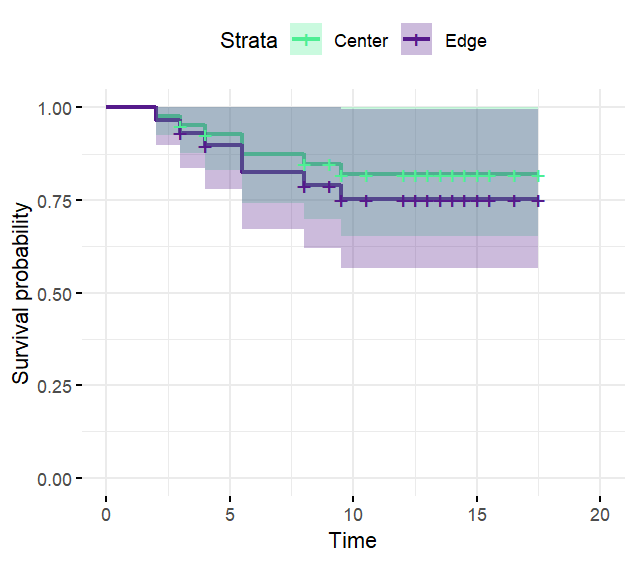


**Figura 4.-** Probabilidad de supervivencia de las puestas control (colocadas en los márgenes de las hojas) y de las puestas experimentales (reubicadas en el centro de la hoja) de *Teratohyla spinosa*. La probabilidad de supervivencia en ambas condiciones disminuyó de manera similar con el tiempo (*p* = 0.6)

Por último, no encontramos evidencia de que la condición, el THI o los huevos por área influyeran en el número de días necesarios para la eclosión completa (Tabla 3).

**Tabla 3-** Resumen de los intervalos de confianza y el valor *p* del modelo MCMC para el tiempo de eclosión.

|  | Menor 95% IC | Mayor 95% IC | *p*MCMC |
| --- | --- | --- | --- |
| Intercepto | -15.56 | 74.28 | 0.4183 |
| Condición (experimento-control) | -0.91 | 0.49 | 0.575 |
| THI | -0.97 | 0.25 | 0.227 |
| Huevos por área | -6.82 | 29.98 | 0.206 |

**Discusión**

Contrario a nuestras expectativas, encontramos que la hidratación y la mortalidad de las puestas no se vieron afectadas por la ubicación en la hoja. Las puestas experimentales y de control no variaron en grosor a lo largo de su desarrollo. Esto sugiere que la hidratación no mejora en las puestas ubicadas en el margen de la hoja y que puede haber diferentes razones que hayan llevado a la evolución de este patrón de oviposición en esta especie. Este hallazgo no fue lo que esperábamos, dada la gran amenaza que representa la desecación para las puestas terrestres/arbóreas [45]. En algunas especies de ranas de cristal, los padres o madres permanecen con sus huevos casi durante todo el período de desarrollo e hidratan activamente sus huevos cuando se sientan sobre ellos (p. ej., *Centrolene peristicta* [46], *Hyalinobatrachium aureoguttatum* [16], *Ikakogi tayrona* [47]). En experimentos de remoción de padres se ha observado que la ausencia del padre cuidador conduce a un aumento considerable en las tasas de mortalidad de la descendencia debido a la deshidratación o la depredación [46,48–50]. Sin embargo, muchas especies de rana de cristal no exhiben un cuidado parental prolongado y sólo se sientan sobre la puesta una vez justo después de la oviposición para aumentar la hidratación de las puestas ricas en gelatina [29,30]; aunque no es el caso de *Espadarana prosoblepon* [26]. Los huevos de *Teratohyla spinosa*, junto con los de otras especies que carecen de un cuidado parental prolongado, están embebidos en una matriz de gelatina altamente absorbente. Se ha propuesto que para estas especies la gelatina puede ser apta para soportar la desecación sin la necesidad de hidratación activa por parte de los padres en condiciones controladas y seminaturales ([34], aunque en este experimento el tamaño de la muestra para *T. spinosa* fue N = 1). Esto es similar a lo que sucede en otras especies de anfibios que producen nidos de espuma [51].

En nuestro estudio, encontramos que en condiciones de campo los embriones no muestran signos de deshidratación probablemente debido a que los niveles de humedad son constantemente altos y hacen que el riesgo de desecación sea muy bajo durante la temporada de lluvias. Se sabe que el estrés hídrico es un impulsor de cambios en la tasa de desarrollo y plasticidad de eclosión en otras especies [19,39,52]. No observamos ningún signo de estrés hídrico durante el desarrollo en nuestro estudio, ya que las puestas de experimento y control no difirieron en los tiempos de eclosión. Asimismo, no hubo indicios de que el estrés hídrico afectara la supervivencia, ya que las tasas de mortalidad no difirieron entre la ubicación de la puesta en las hojas. La ausencia de diferencias significativas en nuestro conjunto de datos no implica automáticamente que la hidratación no haya jugado ningún papel en la evolución del patrón específico de oviposición en nuestra especie de estudio. Una posible razón para esta falta de diferencia podría ser que, aunque no medimos las condiciones climáticas por sitio de oviposición, y hubo cambios considerables diarios y periódicos en la temperatura y la humedad (FNAL, AVA, JC, JGTR, LJ, MGP obs. pers.), estos no fueron lo suficientemente extremos como para afectar las puestas durante todo el experimento, ya que se realizaron durante el pico de la temporada de lluvias [53]. Estas condiciones podrían cambiar especialmente al principio y al final de la temporada de lluvias cuando la humedad es más baja y las precipitaciones son más escasas [54]. Finalmente, las puestas pueden beneficiarse de una mayor hidratación durante los períodos de baja precipitación [55]. Además, las condiciones climáticas altamente impredecibles son más frecuentes debido al cambio climático, lo que hace que los anfibios sean más susceptibles a cambios climáticos extremos [18], especialmente aquellos con reproducción fuera del agua [21]. Por lo tanto, los estudios futuros deben investigar los efectos de las condiciones ambientales más variables en el desarrollo y la supervivencia de las puestas.

Alternativamente, también podría haber otros factores que podrían haber moldeado este patrón de oviposición específico en nuestra especie de estudio. Refsnider y Janzen [1] resumieron los factores que influyen en la selección del sitio de oviposición en seis categorías: 1) maximizar la supervivencia del embrión, 2) maximizar la supervivencia materna, 3) modificar el fenotipo de la descendencia, 4) proximidad a un hábitat adecuado para la descendencia, 5) mantener la filopatría natal y 6) elección indirecta del sitio de oviposición a través de la elección de pareja. En nuestro estudio, no observamos diferencias en la supervivencia de la descendencia o el tiempo de eclosión entre las posiciones de puesta en las hojas. Con respecto a la selección indirecta del sitio a través de la elección de pareja, observamos que los machos llamaban desde las mismas áreas pero diferentes hojas en noches consecutivas, lo que sugiere alguna forma de fidelidad al sitio. Además, observamos que los machos y las hembras se movían sobre varias hojas durante el amplexo. Esto podría indicar una participación activa de ambos sexos en la selección del sitio de oviposición. Al elegir la pareja más adecuada, la hembra selecciona simultáneamente un sitio de oviposición a escala macro. Sin embargo, la decisión de la ubicación exacta para depositar los huevos a escala micro sigue sin estar clara. Por un lado, podría haber beneficios para las hembras en el proceso de puesta de huevos en sí (es decir, podría ser más fácil para ellas poner huevos en los márgenes de las hojas). Por otro lado, también podría haber beneficios para la descendencia, como un mayor éxito de eclosión o frente a la depredación [56]. Estas hipótesis siguen siendo muy especulativos ya que no incluimos dichos factores en nuestro análisis, pero deberían considerarse en estudios futuros.

En resumen, no encontramos evidencia de que la ubicación de la puesta en el márgen de las hojas sea importante para el desarrollo embrionario y el éxito de la eclosión. Nos preguntamos si un efecto podría ser evidente sólo en etapas posteriores del desarrollo o bajo condiciones climáticas más extremas. Alternativamente, la ubicación de las puestas en los márgenes de las hojas podría reflejar algún beneficio para la hembra durante el proceso de puesta de huevos. Por lo tanto, estudios futuros deberían analizar otros posibles beneficios adaptativos de una ubicación particular de la puesta en diferentes especies.

**Agradecimientos**

Queremos agradecer a Edith Villa-Galaviz y Santiago Erazo del proyecto REASSEMBLY por proporcionarnos los datos meteorológicos utilizados en este estudio. También agradecemos a la Fundación Jocotoco, Reserva Canandé, Katrin Krauth, Chiara Correa y al personal de Jocotoco. También queremos agradecer a Christoph Netz, Max Ringler, Oceane LaLoggia y al equipo de Hasli por sus valiosas contribuciones durante el desarrollo de este proyecto.

**Referencias**

1. Refsnider JM, Janzen FJ. Putting eggs in one basket: Ecological and evolutionary hypotheses for variation in oviposition-site choice. Annu Rev Ecol Evol Syst. 2010;41: 39–57. doi:10.1146/annurev-ecolsys-102209-144712

2. Resetarits WJ. Oviposition Site Choice and Life History Evolution 1. 1996. Available: https://academic.oup.com/icb/article/36/2/205/147091

3. Bowyer RT, Kie JG. Effects of scale on interpreting life-history characteristics of ungulates and carnivores. Divers Distrib. 2006;12: 244–257. doi:10.1111/J.1366-9516.2006.00247.X

4. Antoine CM, Forrest JRK. Nesting habitat of ground-nesting bees: a review. Ecol Entomol. 2021;46: 143–159. doi:10.1111/EEN.12986

5. García CG, Lescano JN, Leynaud GC. Oviposition-site selection by *Phyllomedusa sauvagii* (Anura: Hylidae): An arboreal nester inhabiting arid environments. 2013 [cited 29 Jan 2024]. doi:10.1016/j.actao.2013.06.001

6. Pintar MR, Resetarits WJ. Out with the Old, in with the New: Oviposition Preference Matches Larval Success in Cope’s Gray Treefrog, *Hyla chrysoscelis*. Source: Journal of Herpetology. 2017;51: 186–189. doi:10.1670/16-019

7. Touchon JC, Worley JL. Oviposition site choice under conflicting risks demonstrates that aquatic predators drive terrestrial egg-laying. Proceedings of the Royal Society B: Biological Sciences. 2015;282. doi:10.1098/RSPB.2015.0376

8. Hoi H, Krištín A, Valera F, Hoi C. Traditional versus non-traditional nest-site choice: Alternative decision strategies for nest-site selection. Oecologia. 2012;169: 117–124. doi:10.1007/S00442-011-2193-8/FIGURES/3

9. Gould J, Clulow J, Rippon P, Doody JS, Clulow S. Complex trade-offs in oviposition site selection in a cannibalistic frog. Anim Behav. 2021;175: 75–86. doi:10.1016/j.anbehav.2021.02.021

10. Kolbe JJ, Janzen FJ. Impact of nest-site selection on nest success and nest temperature in natural and disturbed habitats. Ecology. 2002;83: 269–281. doi:10.1890/0012-9658

11. Lydersen C, Assmy P, Falk-Petersen S, Kohler J, Kovacs KM, Reigstad M, et al. The importance of tidewater glaciers for marine mammals and seabirds in Svalbard, Norway. Journal of Marine Systems. 2014;129: 452–471. doi:10.1016/J.JMARSYS.2013.09.006

12. Sánchez-Ochoa DJ, Pérez-Mendoza HA, Charruau P. Oviposition site selection and conservation insights of two tree frogs (*Agalychnis moreletii* and *A. callidryas* ). South Am J Herpetol. 2020;17: 17–28. doi:10.2994/SAJH-D-17-00103.1

13. Oh D, Kang J, Song U, Ahn J, Kang C. Oviposition strategies of amphibians in ephemeral streams: a multi-species perspective. 2024 [cited 23 Apr 2024]. doi:10.21203/rs.3.rs-4019240/v1

14. Ortiz-Ross X, Thompson ME, Salicetti-Nelson E, Vargas-Ramírez O, Donnelly MA. Oviposition site selection in three glass frog species. Copeia. 2020;108: 333–340. doi:10.1643/CE-19-243

15. Howard RD. The Influence of Male-Defended Oviposition Sites on Early Embryo Mortality in Bullfrogs. Ecology. 1978;59: 789–798. doi:10.2307/1938783

16. Valencia-Aguilar A, Castro-Herrera F, Patricia M. Microhabitats for oviposition and male clutch attendance in *Hyalinobatrachium aureoguttatum* (Anura: Centrolenidae). Copeia. 2012;2012: 722–731. doi:10.1643/CE-11-173

17. Gould J, Clulow J, Clulow S. High clutch failure rate due to unpredictable rainfall for an ephemeral pool-breeding frog. Oecologia. 2022;198: 699–710. doi:10.1007/S00442-022-05139-2/FIGURES/6

18. Hayden Bofill SI, Blom MPK. Climate change from an ectotherm perspective: evolutionary consequences and demographic change in amphibian and reptilian populations. Biodiversity and Conservation. Springer Science and Business Media B.V.; 2024. pp. 905–927. doi:10.1007/s10531-023-02772-y

19. Nolan N, Hayward M, Callen A, Klop-Toker K. Hydroperiod influences tadpole growth and development in the endangered Littlejohn’s tree frog (*Litoria littlejohni*). Authorea Preprints. 2024 [cited 2 May 2024]. doi:10.22541/AU.171284011.15407463/V1

20. Goldberg J, Quinzio SI, Vaira M. Lack of response to pond desiccation by eggs and tadpoles of the Yungas Red-belly Toad (*Melanophryniscus rubriventris*) in an unpredictable environment. Can J Zool. 2022;100: 296–302. doi:10.1139/CJZ-2021-0164

21. Ringler E, Rojas B, Stynoski JL, Schulte LM. What Amphibians Can Teach Us About the Evolution of Parental Care. Annu Rev Ecol Evol Syst. 2023;54: 43–62. doi:10.1146/ANNUREV-ECOLSYS-102221-050519/CITE/REFWORKS

22. Duellman WE, Trueb L. Biology of Amphibians. Edición 19. Balimore and London: The Johns Hopkins University Press; 1994.

23. Rudolf VHW, Rödel MO. Oviposition site selection in a complex and variable environment: The role of habitat quality and conspecific cues. Oecologia. 2005;142: 316–325. doi:10.1007/s00442-004-1668-2

24. Touchon JC, Warkentin KM. Negative synergism of rainfall patterns and predators affects frog egg survival. Journal of Animal Ecology. 2009;78: 715–723. doi:10.1111/J.1365-2656.2009.01548.X

25. Guayasamin JM, Castroviejo-Fisher S, Trueb L, Ayarzagüena J, Rada M, Vilà C. Phylogenetic systematics of Glassfrogs (Amphibia: Centrolenidae) and their sister taxon *Allophryne ruthveni*. Zootaxa. 2009;2100: 1–97. doi:DOI: 10.11646/zootaxa.2100.1.1

26. Goyes-Vallejos J, Sandoval Siles J, Calero V, Rodriguez N, Machado G. Not enough time: short-term female presence after oviposition does not improve egg survival in the emerald glass frog. Anim Behav. 2024;213: 161–171. doi:https://doi.org/10.1016/j.anbehav.2024.05.008

27. Guayasamin JM, Cisneros-Heredia DF, McDiarmid RW, Peña P, Hutter CR. Glassfrogs of Ecuador: Diversity, Evolution, and Conservation. Diversity 2020, Vol 12, Page 222. 2020;12: 222. doi:10.3390/D12060222

28. Delia JRJ, Ramírez-Bautista A, Summers K. Parents adjust care in response to weather conditions and egg dehydration in a Neotropical glassfrog. Behav Ecol Sociobiol. 2013;67: 557–569. doi:10.1007/S00265-013-1475-Z/FIGURES/4

29. Delia J, Bravo-Valencia L, McDiarmid RW. Notes on paternal behavior in *Hyalinobatrachium* glassfrogs (Anura: Centrolenidae). Phyllomedusa. 2017;16: 101–107. doi:10.11606/issn.2316-9079.v16i1p101-107

30. Delia J, Bravo-Valencia L, Warkentin KM. Patterns of parental care in Neotropical glassfrogs: fieldwork alters hypotheses of sex-role evolution. J Evol Biol. 2017;30: 898–914. doi:10.1111/JEB.13059

31. McDiarmid R. Evolution of Parental Care in Frogs. The development of behavior: comparative and evolutionary aspects. New York: Garland STPM Press; 1978. pp. 127–147.

32. CEPF. Explore the Biodiversity Hotspots | CEPF. 2024 [cited 14 Aug 2024]. Available: https://www.cepf.net/our-work/biodiversity-hotspots

33. Ministerio del ambiente del Ecuador. Sistema de clasificación de los Ecosistemas del Ecuador Continental. Quito; 2012.

34. Delia J, Bravo-Valencia L, Warkentin KM. The evolution of extended parental care in glassfrogs: Do egg-clutch phenotypes mediate coevolution between the sexes? Ecol Monogr. 2020;90: e01411. doi:10.1002/ECM.1411

35. Gosner K. A Simplified Table for Staging Anuran Embryos and Larvae with Notes on Identification. Herpetologica. 1960;16: 183–190.

36. Salazar-Nicholls M-J, del Pino EM. Early development of the glass frogs *Hyalinobatrachium fleischmanni* and *Espadarana callistomma* (Anura: Centrolenidae) from cleavage to tadpole hatching. Number 1 | e88 Amphibian & Reptile Conservation. 2015.

37. REASSEMBLY. 2021 [cited 14 Aug 2024]. Available: https://www.reassembly.de/

38. Mader TL, Davis MS, Brown-Brandl T. Environmental factors influencing heat stress in feedlot cattle,. J Anim Sci. 2006;84: 712–719. doi:10.2527/2006.843712X

39. Warkentin KM. Plasticity of Hatching in Amphibians: Evolution, Trade-Offs, Cues and Mechanisms. Integr Comp Biol. 2011;51: 111–127. doi:10.1093/ICB/ICR046

40. ASAB Ethical Committee/ABS Animal Care Committee. Guidelines for the ethical treatment of nonhuman animals in behavioural research and teaching. Anim Behav. 2023;195: I–XI. doi:10.1016/j.anbehav.2022.09.006

41. Muff S, Nilsen EB, O’Hara RB, Nater CR. Rewriting results sections in the language of evidence. Trends Ecol Evol. 2022;37: 203–210. doi:10.1016/J.TREE.2021.10.009

42. Hadfield JD. MCMC Methods for Multi-Response Generalized Linear Mixed Models: The MCMCglmm R Package. J Stat Softw. 2010;33: 1–22. doi:10.18637/JSS.V033.I02

43. Bürkner P-C. brms: An R Package for Bayesian Multilevel Models using Stan. 2017.

44. Therneau TM. Package “survival.” The Comprehensive R Archive Network. 2024. Available: https://github.com/therneau/survival

45. González K, Warkentin KM, Güell BA. Dehydration-induced mortality and premature hatching in gliding treefrogs with even small reductions in humidity. Ichthyology & Herpetology. 2021;109: 21–30. doi:10.1643/H2020085

46. Salgado AL, Guayasamin JM. Parental care and reproductive behavior of the minute dappled glassfrog (Centrolenidae: *Centrolene peristictum*). South Am J Herpetol. 2018;13: 211–219. doi:10.2994/SAJH-D-17-00066.1

47. Valencia LB, Delia J. Maternal care in a glassfrog: care function and commitment to offspring in *Ikakogi tayrona*. Behav Ecol Sociobiol. 2016;70: 41–48. doi:10.1007/s00265-015-2022-x

48. Vockenhuber EA, Hödl W, Amézquita A. Glassy Fathers Do Matter: Egg Attendance Enhances Embryonic Survivorship in the Glass Frog *Hyalinobatrachium valerioi*. Source: Journal of Herpetology. 2009.

49. Valencia-Aguilar A, Domingos &, Rodrigues J, Prado CPA. Male care status influences the risk-taking decisions in a glassfrog. Behav Ecol Sociobiol. 2020; 74–84. doi:10.1007/s00265-020-02869-2

50. Chaves-Acuña W, Salazar-Zúñiga JA, Chaves G. Egg clutch survival under prolonged paternal care in a glass frog, *Hyalinobatrachium talamancae*. Copeia. 2020;108: 514–521. doi:10.1643/CE-19-322

51. Gould J, Valdez J, Clulow J, Clulow S. Left High and Dry: Froth Nesting Allows Eggs of the Anuran Amphibian to Complete Embryogenesis in the Absence of Free-Standing Water. https://doi.org/101643/h2020142. 2021;109: 537–544. doi:10.1643/H2020142

52. Delia J, Rivera-Ordonez JM, Salazar-Nicholls MJ, Warkentin KM. Hatching plasticity and the adaptive benefits of extended embryonic development in glassfrogs. Evol Ecol. 2019;33: 37–53. doi:10.1007/s10682-018-9963-2

53. Touchon JC, McMillan WO, Ibáñez R, Lessios HA. Flexible oviposition behavior enabled the evolution of terrestrial reproduction. Proc Natl Acad Sci U S A. 2024;121: e2312371121. doi:10.1073/PNAS.2312371121/FORMAT/EPUB

54. INAMHI. Aplicaciones Web. In: GeoGlows INAMHI [Internet]. 2025 [cited 20 Jan 2025]. Available: https://servicios.inamhi.gob.ec/aplicaciones-web/

55. Lehtinen RM, Green SE, Pringle JL. Impacts of paternal care and seasonal change on offspring survival: A Multiseason experimental study of a caribbean frog. Ethology. 2014;120: 400–409. doi:10.1111/eth.12215

56. Kumar R, Muhid P, Dahms HU, Tseng LC, Hwang JS. Potential of three aquatic predators to control mosquitoes in the presence of alternative prey: A comparative experimental assessment. Mar Freshw Res. 2008;59: 817–835. doi:10.1071/MF07143

**Material Suplementario**

**S1. Texto-** Priors utilizados en los análisis

**S2. Texto-** Versión en español del manuscrito
